# Brackish Water Desalination Using Electrodialysis: Influence of Operating Parameters on Energy Consumption and Scalability

**DOI:** 10.3390/membranes15080227

**Published:** 2025-07-31

**Authors:** Angie N. Medina-Toala, Priscila E. Valverde-Armas, Jonathan I. Mendez-Ruiz, Kevin Franco-González, Steeven Verdezoto-Intriago, Tomas Vitvar, Leonardo Gutiérrez

**Affiliations:** 1 Faculty of Engineering in Earth Sciences, ESPOL Polytechnic University, Campus Gustavo Galindo, Km. 30.5 Vía Perimetral, Guayaquil 090902, Ecuador; angnmedi@espol.edu.ec (A.N.M.-T.); priesval@espol.edu.ec (P.E.V.-A.); jonimend@espol.edu.ec (J.I.M.-R.); krfranco@espol.edu.ec (K.F.-G.); sjverdez@espol.edu.ec (S.V.-I.); tvitvar@espol.edu.ec (T.V.); 2 Department of Geography, Faculty of Science, Humanities and Education, Technical University of Liberec, Komenského 2, 46005 Liberec, Czech Republic; 3 Centre for Advanced Process Technology for Urban Resource Recovery (CAPTURE), Particle and Interfacial Technology Group, Ghent University, Frieda Saeysstraat 1, 9052 Ghent, Belgium; 4 PaInt, Particle and Interfacial Technology Group, Department of Green Chemistry and Technology, Faculty of Bioscience Engineering, Ghent University, Coupure Links 653, 9000 Ghent, Belgium; 5 Facultad del Mar y Medio Ambiente, Universidad Del Pacifico, Guayaquil 090112, Ecuador

**Keywords:** groundwater, electrochemical, membrane separation, specific energy consumption, electrodialysis

## Abstract

Groundwater is one of the main water sources for consumption, domestic use, agriculture, and tourism in coastal communities. However, high total dissolved solids (TDS) levels in the water (700–2000 mg L^−1^ TDS) and electrical conductivity (3000–5000 µS cm^−1^) threaten the health and economic growth opportunities for residents. This research aims to evaluate the performance of a laboratory-scale electrodialysis system as a technology for desalinating brackish water. For this purpose, water samples were collected from real groundwater sources. Batch experiments were conducted with varying operational parameters, such as voltage (2–10 V), feed volume (100–1600 mL), recovery rate (50–80%), and cros-flow velocity (1.3–5.1 cm s^−1^) to determine the electrodialysis system setup that meets the requirements for drinking water in terms of TDS and energy efficiency. A total specific energy consumption of 1.65 kWh m^−3^, including pumping energy, was achieved at a laboratory scale. The conditions were as follows: flow velocity of 5.14 cm s^−1^, applied voltage of 6 V, feed volume of 1.6 L, and a water recovery of 66%. Furthermore, increasing the flow velocity and the applied voltage enhanced the desalination kinetics and salt removal. Additionally, the system presented opportunities for scalability. This research aims to evaluate a sustainable membrane-based treatment technology for meeting the growing demand for water resources in coastal communities, particularly in developing countries in South America.

## 1. Introduction

Coastal communities often rely on groundwater as their primary source of drinking water, domestic use, agriculture, and tourism [1,2,3]. However, the salinity of these groundwater resources is frequently influenced by factors such as the geochemical evolution of the aquifer, the marine origin of the sediments, and seawater intrusion [4,5]. Hydrochemical studies of coastal aquifers worldwide have reported electrical conductivity (EC) values ranging from 1700 to 5000 µS cm^−1^ [6,7,8], levels that often exceed the organoleptic thresholds for human consumption. Elevated salinity not only makes water unsuitable for drinking but has also been associated with increased risks of cardiovascular, respiratory, gastrointestinal, and dermatological conditions [6,7]. The World Health Organization (WHO) recommends EC levels below 800 µS cm^−1^ for potable water [8], which aligns with the Ecuadorian NTE INEN 1108 Technical Standard (https://www.normalizacion.gob.ec/) that establishes the quality requirements for drinking water for human consumption, including physical, chemical, microbiological, and radioactive parameters (i.e., Unified Text of Secondary Legislation of the Ministry of the Environment). In this context, the development and implementation of sustainable desalination technologies are crucial to ensuring access to drinking water for coastal populations.

Among the available desalination technologies, membrane-based processes are particularly popular, operating through pressure, temperature, concentration, or electrical potential gradients to remove salts and other dissolved contaminants [9,10,11]. Reverse osmosis (RO) and electrodialysis (ED) are two of the most extensively studied membrane-based processes in the scientific literature. ED has shown notable advantages over RO for treating brackish water within the EC range of 1600–7800 µS cm^−1^ [12,13]. For example, one study reported that ED required 35% less energy than RO when treating water with an EC of 4000 µS cm^−1^, although this trend reversed at higher concentrations, where RO outperformed ED with 27% lower consumption [14]. Additional research has shown that ED achieved energy savings of 30%, 50%, and 75% over RO at EC levels of 4700, 3200, and 1600 µS cm^−1^, respectively [15]. Moreover, the capital cost for a 1000 L h^−1^ desalination plant was estimated at USD 6400 for RO compared to USD 3200 for ED at approximately 3 mS cm^−1^ [16]. In trials using synthetic brackish water with an electrical conductivity (EC) of 7800 µS cm^−1^, ED achieved a lower specific energy consumption (SEC) of 0.3 kWh m^−3^, compared to 4 kWh m^−3^ for RO, reinforcing its potential for decentralized domestic applications [12]. In addition to its energy efficiency, ED offers operational advantages such as higher membrane tolerance to residual chlorine, elevated temperatures, and microbial contamination when compared to RO systems [17]. Furthermore, ED membranes have demonstrated longer operational lifespans (7–10 years) [18], higher water recovery rates (80–90%) [19], and low SEC values (0.3–1.5 kWh m^−3^) [20,21] while operating at low pressures (0.2–0.4 bar), which is particularly advantageous for decentralized applications [12,17]. ED systems can also be powered by photovoltaic sources, enabling deployment in vulnerable communities without the need for high-voltage infrastructure. For instance, small-scale PV configurations of 33 W and 12 V have achieved over 99% salt removal, while larger installations (e.g., Fukue plant, Japan) have reached up to 200 m^3^/day with energy demands ranging from 0.6 to 1 kWh/m^3^ [22].

Laboratory-scale studies have explored how operational variables, including applied voltage, initial salinity, and flow rate, affect ED performance [23,24,25,26]. Sadrzadeh et al. identified initial salinity as the most influential factor in desalination efficiency (82.4%), followed by applied voltage (8.5%), flow rate (7.5%), and temperature (1.5%) [26]. In a related study, current efficiency was assessed across voltages ranging from 2 to 6 V, flow rates between 0.1 and 5.0 mL s^−1^, and feed EC values from 7.8 to 46.8 mS cm^−1^. Results indicated that current usage was more efficient at flow rates below 1 mL s^−1^ and at lower initial salinities [25]. Desalination kinetics were also found to be slower for real groundwater samples (EC 7800 µS cm^−1^) compared to synthetic analogs, highlighting the influence of complex real water matrices on ED performance [24]. Pilot-scale evaluations, primarily conducted in the Middle East, Asia, Europe, and North America, have focused on energy consumption under varying operating conditions. For example, an ED system with 10 membrane pairs reduced the EC below 800 µS cm^−1^, achieving SEC values between 1.0 and 1.5 kWh m^−3^ for feed salinities of 3200 to 16,000 µS cm^−1^. Another configuration, utilizing 24 membrane pairs, achieved 95% salt removal from groundwater (1600–7800 µS cm^−1^) at a flow rate of 130 mL min^−1^ [27]. Similarly, a system required 2.1 kWh m^−3^ to desalinate water with a 4 g/L TDS [28]. Owing to its relatively low energy demands, ED systems have also been successfully powered by solar energy, proving viable in off-grid coastal communities [27,28,29,30].

Despite these technological advancements, the application of ED in South American coastal communities remains limited. In many of these communities, untreated brackish water remains the primary source of drinking water due to the lack of treatment infrastructure, often hindered by high capital costs, complex operational requirements, and dependence on grid electricity [3,31,32]. Addressing these challenges requires the development of low-cost, energy-efficient, and adaptable solutions suited for decentralized deployment. In this context, the present study evaluated the performance of a laboratory-scale ED system designed to desalinate real brackish groundwater collected from coastal communities in Ecuador, where the EC typically ranges between 3000 and 5000 µS cm^−1^. This salinity has been identified as favorable for energy-effective desalination, suggesting that ED may offer a robust alternative, particularly when integrated with renewable energy sources. This study specifically assessed the influence of applied voltage on desalination kinetics and energy consumption, aiming to optimize salt removal performance while minimizing treatment time and energy consumption. Target salt removal efficiencies (30% to 70%) and compliance with WHO drinking water standards (EC < 800 µS cm^−1^) and the Ecuadorian Ecuadorian NTE INEN 1108 Technical Standard that establishes the quality requirements for drinking water for human consumption (i.e., Unified Text of Secondary Legislation of the Ministry of the Environment) were evaluated alongside water recovery through theoretical and experimental comparisons. Additionally, system scalability was investigated by analyzing the effects of feed volume, EC, and flow rate on treatment efficiency. These findings contribute to the design of a decentralized, cost-effective desalination system tailored to resource-constrained coastal settings [33].

## 2. Materials and Methods

### 2.1. Study Area, Field Sampling, and Chemical Analysis of Water Samples

**Figure 1:**
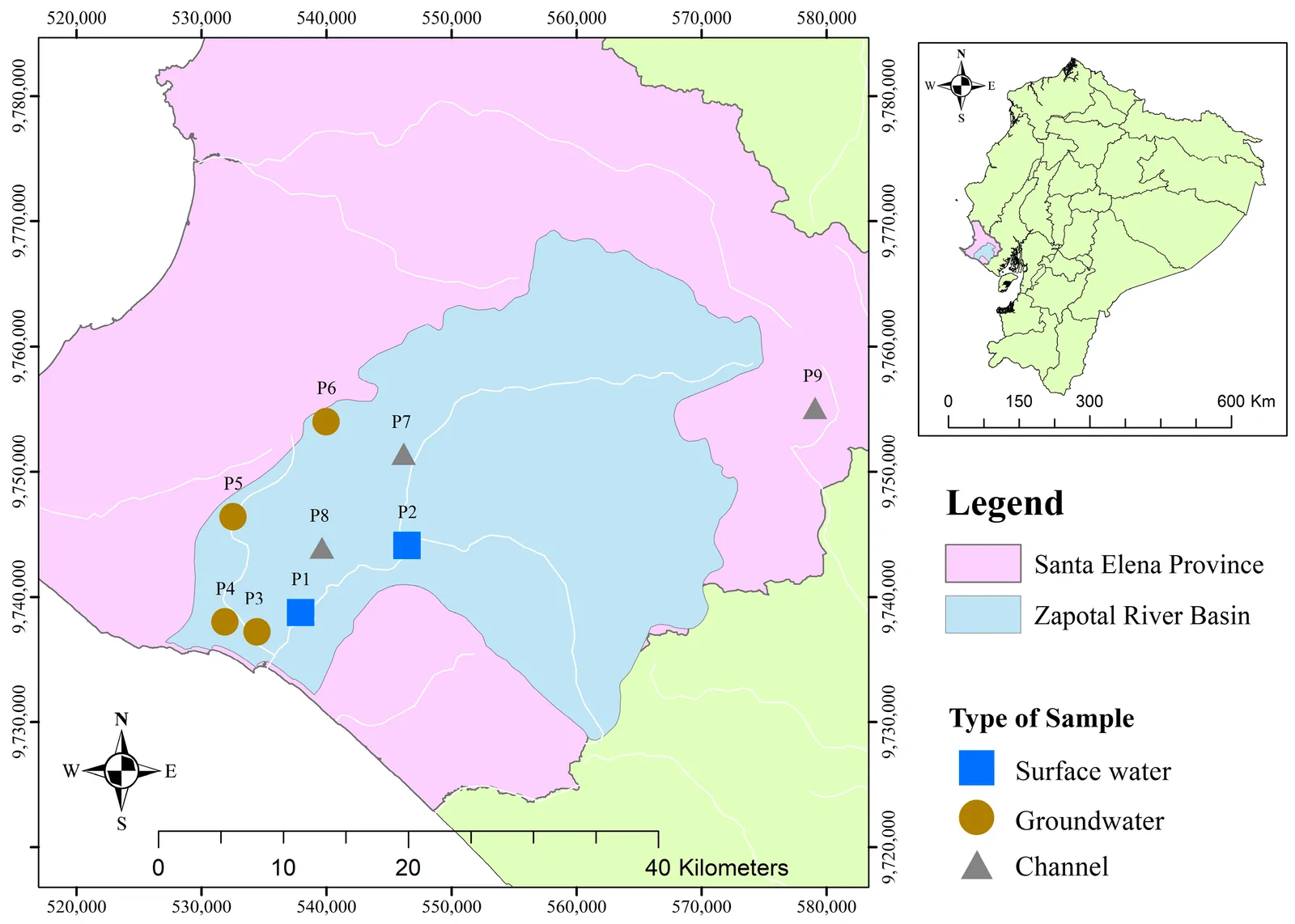
Map of sampling location: Zapotal River Basin, Santa Elena Province.

Physicochemical parameters, including pH, temperature, electrical conductivity (EC), total dissolved solids (TDS), and turbidity, were measured in the field using HQ40d portable multiparameter probes (HACH, Loveland, CO, USA) and a 2100Q turbidity meter (HACH ,Loveland, CO, USA), with an accuracy of ±0.5 for EC-TDS, ±0.3 °C for temperature, ±0.02 for pH, and ±2% of reading plus stray light for the 2100Q- HACH. The study area was the Zapotal River Basin, where seven sampling locations were selected based on accessibility, as shown in Figure 1. A total of 200 L of water was collected to encompass a wide range of conductivities within the study area. The samples were stored at 4 °C to preserve their chemical integrity until further analysis.

The concentrations of major ions, including Cl^−^, HCO_3_^−^, NO_3_^−^, Na^+^, K^+^, SO_4_^2−^, CO_3_^2−^, Ca^2+^, and Mg^2+^, were determined using titration, ultraviolet-visible (UV-Vis) spectrophotometry (DR 3900, HACH), and potentiometry. Bicarbonate, calcium, and total hardness concentrations were quantified using buret titration methods (HACH methods 8221, 8222, and 8226, respectively). Nitrate, nitrite, sulfate, chloride, and potassium concentrations were measured using the cadmium reduction (HACH method 8039), ferrous sulfate (HACH method 8153), USEPA SulfaVer 4 (HACH method 8051), mercuric thiocyanate (HACH method 8113), and tetraphenylborate (HACH method 8049) methods, respectively. Sodium concentration was determined using the direct ion-selective electrode (ISE) potentiometric method (HACH method 8322).

### 2.2. Lab-Scale Electrodialysis Setup

The experimental setup of the lab-scale ED system consisted of a peristaltic pump, a DC power supply with integrated user–interface software for data acquisition and operation control, a multiparameter sensor for EC monitoring, and an electrochemical stack.

A BTF Dispense peristaltic pump (Landto Tech, Baoding Ding, Hebei Province, China) equipped with YZ15 pump heads, configured for parallel flow, circulated groundwater samples through each compartment of the electrochemical stack (i.e., concentrated and diluted streams). Additionally, a Na_2_SO_4_ solution (7.0 mM, EC of ~1500 μS cm^−1^) was circulated as the electrolyte. The system’s energy input was supplied by a CS150DC power source (CorrTest, Wuhan, Hubei Province, China) operating in potentiostatic mode (i.e., at constant voltage). Energy consumption was monitored using CS Studio5 software version 5.5, which facilitated data acquisition and operational control. A HQ40d portable multiparameter instrument (HACH, Loveland, CO, USA) with an EC sensor was placed in the dilute compartment for real-time conductivity measurements.

The electrochemical stack comprised an anode, a cathode, and ion exchange membranes, including cation-exchange membranes (CEM) and anion-exchange membranes (AEM). The anode consisted of a Ru-Ir mesh, while the cathode was a solid stainless-steel plate. The stack contained six ion-exchange membrane pairs (Fujifilm CEM/AEM type II, 64 cm^2^ active area).

### 2.3. Experimental Procedure

**Figure 2:**
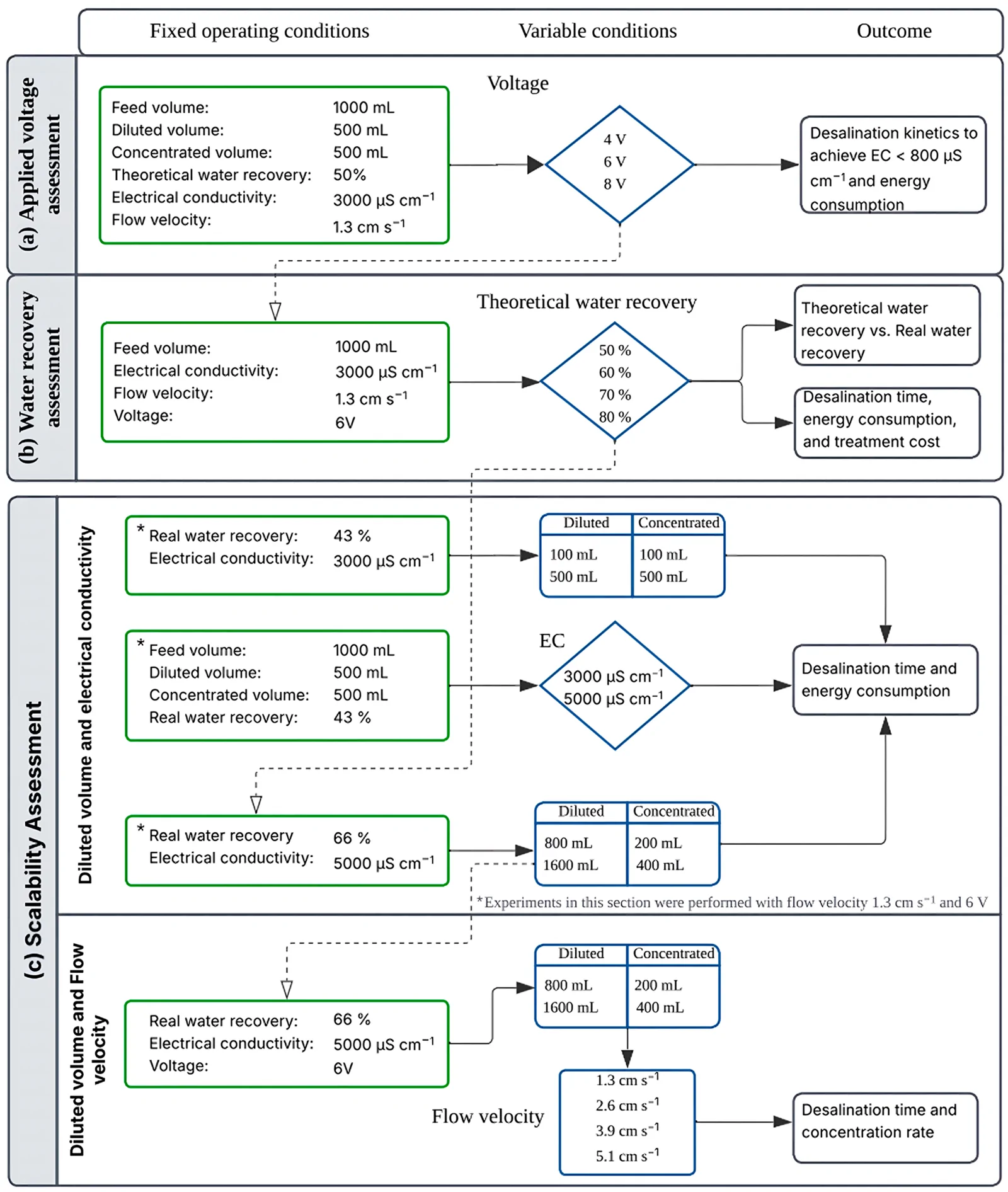
Scheme of the experimental procedure showing (**a**) the applied voltage, (**b**) the water recovery, and (**c**) the scalability assessment.

Figure 2 summarizes the experimental procedure, which was structured into three main variables to study: (a) applied voltage, (b) water recovery, and (c) scalability.

First, the effect of applied voltage was evaluated under potentiostatic conditions at 4, 6, and 8 V to analyze the desalination kinetics required to achieve EC levels suitable for domestic water consumption (e.g., <800 μS cm^−1^). Additionally, energy consumption was recorded at these voltages to determine the electrochemical specific energy consumption (i.e., termed SEC) for different salt removal efficiencies (30%, 40%, 50%, 60%, and 70%). Additionally, the specific energy consumption resulting from the pumping of solutions was calculated based on flow rates, pressure drop, and efficiency, and was termed SECp. The total specific energy consumption (termed SECt) comprised SEC and SECp. The fixed experimental conditions for these tests included a feedwater volume of 1000 mL, comprising 500 mL of diluted water (i.e., treated water with a reduced salt concentration for consumption purposes), and 500 mL of concentrated water, corresponding to a theoretical water recovery (TWR) of 50%. This theoretical water recovery does not account for water transport during the desalination process. The initial EC of feedwater was 3000 μS cm^−1^, and the flow velocity was maintained at 1.3 cm s^−1^.

Subsequently, the effect of TWR on desalination time, specific energy consumption (SEC), and treatment cost was analyzed. The real water recovery (RWR), which accounts for water transport during the desalination process, was measured at TWR levels of 50%, 60%, 70%, and 80% using an electronic scale. The EC of the groundwater sample (e.g., 3000 μS cm^−1^), the flow velocity of 1.3 cm s^−1^, and an applied voltage of 6 V were maintained as fixed experimental parameters.

Next, the scalability of the ED system was systematically assessed by varying the volume of diluted water and increasing the initial EC of the feedwater. These experimental conditions are detailed in Figure 2 in the scalability assessment section. The effect of increasing the diluted volume from 100 to 500 mL on desalination time and energy consumption was evaluated while maintaining an RWR of 43%, an initial EC of 3000 μS cm^−1^, and a flow velocity of 1.3 cm s^−1^ at 6 V. The effect of increasing the initial EC of groundwater from 3000 to 5000 μS cm^−1^ was examined while maintaining an RWR of 43%, a diluted volume of 500 mL, an applied voltage of 6 V, and a flow velocity of 1.3 cm s^−1^. The impact of increasing the diluted volume from 800 to 1600 mL was assessed under conditions of 66% RWR, an initial EC of 5000 μS cm^−1^, a flow velocity of 1.3 cm s^−1^, and an applied voltage of 6 V.

The scalability assessment also included an evaluation of the effect of increasing the flow velocity from 1.3 to 2.6, 3.9, and 5.1 cm s^−1^ on desalination time to achieve a final EC < 800 μS cm^−1^. Finally, the impact of increasing the diluted volume from 800 to 1600 mL at different flow velocities was analyzed to determine the influence of flow rate on desalination and concentration rates. Experiments were conducted in two ED stacks running in parallel to assess the replicability of the experiments. However, only data from one stack were presented in the current study based on the consistent trends observed.

## 3. Results and Discussion

### 3.1. Hydrochemical Characterization of the Brackish Water

As a first step, a rigorous water characterization was performed for the seven selected locations. Table 1 presents the water samples and their corresponding physicochemical parameters, as measured in the field. The pH ranged from 7.2 to 8.5, which falls within the drinking water standards recommended by the WHO and EPA [34,35,36]. Groundwater and surface water exhibited EC values between 1219 and 3250 μS cm^−1^, with corresponding TDS concentrations ranging from 564 to 1709 mg L^−1^. These variations in EC are likely influenced by the geological formations existing in the study area. For instance, the Zapotal River Basin is associated with the Tablazo formation, which is composed primarily of sedimentary rocks derived from marine sediment accumulation due to its proximity to the Pacific Ocean. The turbidity levels of groundwater samples varied from 0.4 to 2.5 NTU.

Water samples from the artificial Chongon and San Vicente channels presented lower EC (109–146 μS cm^−1^) and TDS (49–65 mg L^−1^). These channels transport surface water from the Daule River Basin to communities in the Zapotal River Basin for irrigation purposes. Turbidity levels of 4.8 and 10.5 NTU were observed, likely influenced by organic matter, suspended clays, microplankton, or pollutants from domestic activities.

**Figure 3:**
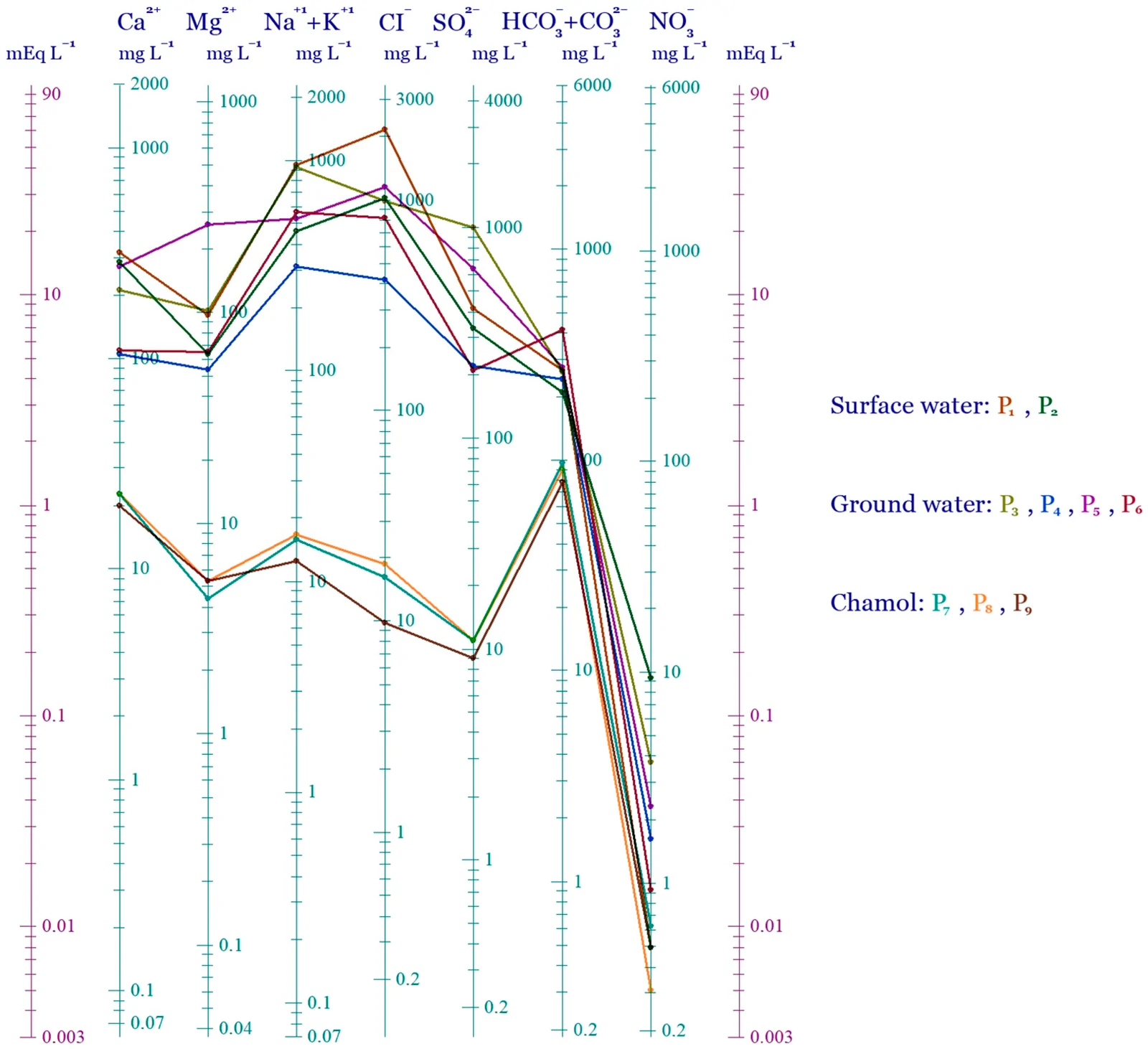
Schöeller-Berkaloff diagram of water collected samples.

Figure 3 presents the Schöeller-Berkaloff diagram for surface water (P1–P2), groundwater (P3–P6), and channel water (P7–P9). The hydrochemical composition of surface water and groundwater exhibited similarities, with high concentrations of Ca^2+^, Mg^2+^, Na^+^, K^+^, Cl^−^, SO_4_^2−^, and HCO_3_^−^, suggesting that both sources are influenced by the mineralogy of the local geological formation. In contrast, channel water, originating from a different basin with distinct hydrochemical characteristics, presented lower ion concentrations. Across sampling points P1 to P6, Na^+^ and K^+^ concentrations ranged from 250 to 950 mg L^−1^, Cl^−^ from 400 to 2000 mg L^−1^, and SO_4_^2−^ from 200 to 1000 mg L^−1^, exceeding WHO drinking water guidelines, which set limits of 200 mg L^−1^ for Na^+^ and Cl^−^ and 250 mg L^−1^ for SO_4_^2−^. Ca^2+^ and Mg^2+^ concentrations ranged from 100 to 310 and 50 to 260 mg L^−1^, respectively. These ion concentrations may be attributed to aquaculture activities, where discharges infiltrate the soil and contribute to groundwater salinity.

Conversely, the lower mineral ion concentrations in channel water may result from its origin outside the local aquifers, with concrete-lined irrigation channels limiting interaction with the geological formation. Based on its EC of ~3000 μS cm^−1^, accessibility, and relatively low turbidity (1.50 NTU), groundwater from sampling point P3 was selected for ED desalination. The low turbidity minimizes the risk of organic fouling in the ED membranes. Furthermore, this groundwater source currently serves as a domestic water supply for the local community.

### 3.2. Electrodialysis

#### 3.2.1. Effect of Voltage on Desalination Kinetics and Specific Energy Consumption

**Figure 4:**
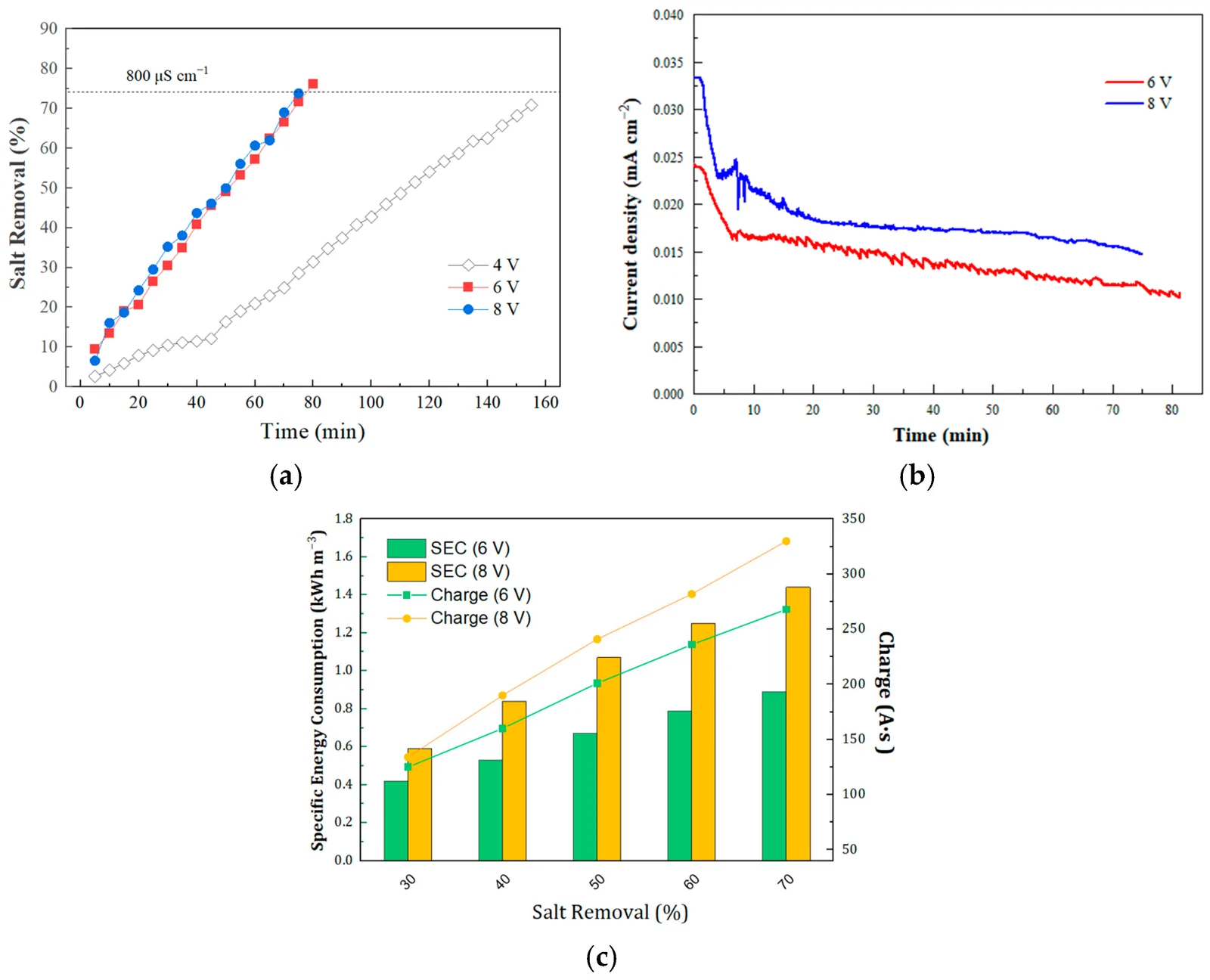
Effects of voltage on: (**a**) salt removal efficiencies (%) and desalination kinetics (min) (95% confidence and prediction bands shown in Figure A1), (**b**) current density, and (**c**) SEC and charge at different salt removal efficiencies. Groundwater samples with an initial EC of ~3000 µS cm^−1^ were used as the feed. A feed volume of 1000 mL was processed, with 500 mL in the diluted compartment and 500 mL in the concentrate compartment, corresponding to a theoretical water recovery of 50%. The solution samples were circulated at a cross-flow velocity of 1.3 cm s^−1^.

Figure 4a shows the effect of increasing voltage on desalination kinetics to achieve an EC < 800 µS cm^−1^, corresponding to salt removal efficiencies exceeding 76%, which are suitable for drinking water purposes. As the applied voltage increased from 4 to 6 and 8 V, the salt removal efficiency improved from 8%, 20%, and 25%, respectively, within the first 20 min. At 4 V, the target EC was not reached even after 155 min of operation; under this voltage condition, the salt removal efficiency was 71%. In contrast, at 6 and 8 V, the desalination time decreased to 80 and 75 min, respectively, achieving the target EC in the diluted compartment. These results indicate that higher voltages enhance salt removal efficiency and reduce desalination time. For example, after 75 min of operation, salt removal efficiencies of 29%, 72%, and 74% were observed at 4, 6, and 8 V, respectively. Notably, the 8 V system shortened the operation time by only 5 min compared to the 6 V system, suggesting that increasing the voltage beyond 6 V does not provide a significant operational advantage in terms of desalination time. Several studies have explored the role of voltage under similar recovery conditions (e.g., TWR of 50%). Demircioglu et al. investigated an ED system using voltages ranging from 3 to 15 V with 10 membrane pairs, achieving a salt removal of nearly 90% after 18 min at 10 V. However, increasing the voltage to 15 V did not further reduce the desalination time [37]. Sadrzadeh et al. reported salt removal efficiencies of approximately 60%, 80%, and 99% at 5, 7, and 9 V, respectively, in an ED system utilizing a single membrane pair [26]. Similarly, Nyguyen et al. applied voltages between 12 and 16 V in an ED system treating 1.6 L with 16 pairs of membranes. While 90 min of operation at 12 V was insufficient to reach an EC < 800 μS cm^−1^, applying 14 and 16 V resulted in desalination times of 55 and 38 min, respectively [23]. Banasiak et al. evaluated an ED system treating feedwater with an EC of 7800 μS cm^−1^ and determined that 12 V was sufficient to reach 800 μS cm^−1^ within 50 min [24]. These studies collectively indicate that low voltages result in longer desalination times and extended operational times. However, recent studies on ED stack optimization have shown that the optimal voltage for brackish water desalination depends on factors such as salinity and water recovery. Specifically, the optimal voltage decreased as salinity increased, as long as water recovery exceeded 50% [38].

Figure 4b presents the current density profile of the ED system under potentiostatic conditions. Experiments at 4 V were excluded because they failed to achieve the target EC (<800 µS cm^−1^) after 150 min of operation. The initial current densities at 6 and 8 V were 0.02 and 0.03 mA cm^−2^, respectively. To reach an EC ~800 µS cm^−1^, the SEC was 0.96 and 1.51 kWh m^−3^ for the 6 and 8 V systems, respectively. The lowest SEC was achieved at 6 V after 80 min of operation, resulting in a salt removal efficiency of 76%. These findings suggest that the system is approaching its efficiency limit at 8 V. It has been reported in the literature that increasing the voltage beyond a certain limit does not enhance ion migration efficiency; rather, it can lead to ion accumulation in the interfacial region, increasing charge density in the diffusion boundary layer and inducing concentration polarization [39,40]. Similarly, Chen et al. reported that when the limiting current density is surpassed, the salt flux decreases due to charge carrier depletion, resulting in a decrease in ionic transport into the diluted channel [41]. Consequently, the electrical resistance of the solution increases, and as a compensatory mechanism, water molecule dissociation may occur [42]. Nikonenko et al. identified water splitting as a key mechanism, where the generation of H^+^ and OH^−^ ions in the depleted diffusion layer enhances counterion binding, i.e., H^+^ attracts anions and OH^-^ attracts cations from the solution, thereby hindering effective ion migration through the membrane due to fouling, scaling, and membrane deterioration [39,43].

The limiting current density was theoretically estimated at 0.2 mA cm^−2^, a value higher than that observed experimentally. This discrepancy arises because the idealized calculation does not account for the complex ionic composition (Ca^2+^, Mg^2+^, SO_4_^2−^, HCO^3−^), where divalent ions like Ca^2+^ compete with and displace Na^+^ during transport. Additionally, the model does not account for current leakage, resulting in an underestimation of the actual ionic transport resistance [44]. According to the estimated limiting current density, the production of H^+^ at the membrane–solution interface (i.e., due to water splitting) is negligible when the system operates below the limiting current regime [45]. However, H^+^ can still be generated in the electrolyte channels near the electrodes due to electrochemical redox reactions [46].

Figure 4c illustrates the effect of salt removal efficiency on SEC. The results show that achieving the same salt removal efficiency required a higher SEC as the applied voltage increased. For example, at 30% salt removal, the SEC increased from 0.4 kWh m^−3^ at 6 V to 0.6 kWh m^−3^ at 8 V. A similar trend was observed at 50% salt removal, where the SEC increased from 0.7 at 6 V and 1.1 kWh m^−3^ at 8 V. At 70% salt removal, the SEC reached 0.9 kWh m^−3^ at 6 V and further increased to 1.4 kWh m^−3^ at 8 V. These findings align with those reported by Patel et al. [21], which suggests that as salt removal increases, ion depletion in the diluted compartment of the solution leads to a decrease in ionic strength. This depletion is associated with a potential drop, requiring higher driving forces to maintain ion transport, thereby increasing energy consumption. In addition, Figure 4c shows the effect of salt removal efficiency on charge requirements within the system, demonstrating that higher salt removal demands higher charges. Specifically, achieving 30% salt removal required a charge of 125 A∙s at 6 V, while at 8 V, it increased to 134 A∙s. To achieve 70% salt removal, the charge demand increased to 268 and 330 A∙s for the 6 V and 8 V systems, respectively. These results confirm that higher applied voltages result in increased energy consumption due to the greater energy needed to overcome the high ionic resistance of the solution. Moreover, the enhanced co-ion transport required to maintain membrane electroneutrality contributes to more pronounced concentration gradients across the transmembrane region, further increasing energy demand [12,21]. Additionally, it is possible that at this high voltage (i.e., 8 V, which induces the total cell voltage to exceed the sum of membrane and solution resistances) would cause a hydrogen evolution reaction (HER) overpotential.

Among the experimental conditions tested, the 6 V system showed the best results in terms of SEC, kinetics, and salt removal rate, and thus was selected for the next phase of experiments. This outcome is consistent with the insights of Rubinstein et al., who reported that the limiting current density for a membrane pair in a conventional ED system is expected to be close to 1 V, consistent with several previous investigations [47].

#### 3.2.2. Impact of Water Recovery on Desalination Kinetics and Treatment Cost

**Figure 5:**
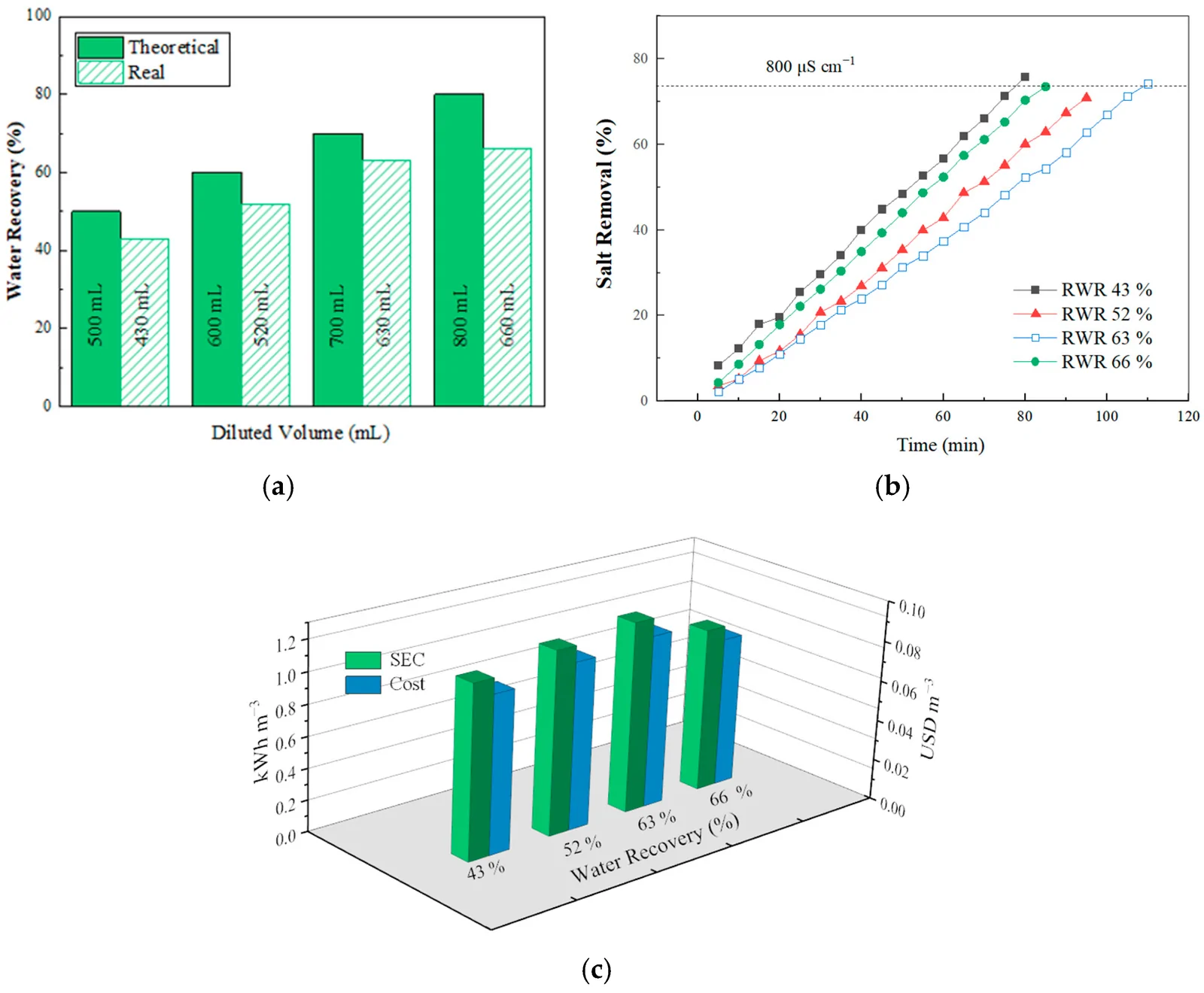
(**a**) TWR vs. measured RWR monitoring in the diluted compartment. (**b**) Effect of RWR on desalination time and salt removal efficiency (95% confidence and prediction bands shown in Figure A1). (**c**) Influence of RWR on SEC and treatment cost. The feedwater exhibited an initial EC of ~3000 µS cm^−1^, and experiments were conducted at a fixed cross-flow velocity of 1.3 cm s^−1^ with an applied voltage of 6 V.

Figure 5a presents the comparison between TWR and RWR in the diluted compartment during the ED experiments, considering TWR values of 50%, 60%, 70%, and 80%. Due to water transport phenomena occurring during the process, the corresponding RWR values were measured as 43%, 52%, 63%, and 66%, respectively. Three mechanisms primarily drive water transport: (i) hydrostatic pressure differences across the membrane, (ii) osmotic transport induced by concentration gradients between the solutions separated by the membrane, and (iii) electro-osmosis, in which water molecules within the hydration shell of migrating ions are carried across the membrane under an electric potential gradient [48,49].

Figure 5b illustrates the relationship between RWR and desalination time. As the RWR increased from 43% to 52% and 63%, the operating time increased from 80 to 110 min, consistent with the trend reported by Yan et al. [50]. However, when the RWR exceeded 63% (e.g., RWR of 66%), the desalination time decreased to 85 min. A similar effect was observed by Sun et al. [51], who evaluated water recovery from 50% to 75% in a two-stage ED system with five membrane pairs per stage, achieving an average salt removal efficiency of 70%. Their results indicated that increasing water recovery from 50% to 75% reduced the desalination time from 40 to 25 min to reach an EC below 800 µS cm^−1^. These findings suggest that higher water recovery reduces the concentration gradient and mitigates the back diffusion of salts from the concentrate to the diluted stream, thereby enhancing ion migration and accelerating the desalination process.

Figure 5c highlights the relationship between RWR, SEC (i.e., solely comprising electrochemical energy, Section 2.3), and treatment costs in the ED system. The energy costs were estimated based on the electricity price per kWh according to Ecuador’s public energy tariff (i.e., USD 0.092/kWh). At an RWR of 43%, the SEC was 1.1 kWh m^−3^, corresponding to a cost of 0.10 USD m^−3^. As the RWR increased to 63% and 66%, the SEC values reached 1.2 and 1.0 kWh m^−3^, respectively, with corresponding costs of 0.11 and 0.09 USD m^−3^. These results underscore the inverse correlation between SEC and RWR, indicating that higher water recovery rates are associated with reduced energy consumption. Although a reduction in SEC implies lower energy consumption, it often requires a greater number of cell pairs, thereby increasing the capital expenditure (CAPEX). However, other studies have reported that operating at larger treatment scales can significantly reduce operating costs (OPEX); for instance, scaling up from 500 to 10,000 m^3^/d has been shown to lower OPEX from 1.85 to 0.86 USD/m^3^ [52]. Specifically, increasing the recovery rate from 43% to 66% resulted in a 5% decrease in energy consumption. These findings were supported by Bi et al., who observed that at low conductivities, increasing water recovery had little effect on SEC. In contrast, at conductivities exceeding 6000 µS/cm, SEC rose with increasing water recovery, significantly raising the associated cost [38].

Several authors have suggested that increasing water recovery beyond 50% promotes convective water transport through the IEMs, leading to salt accumulation in the concentrate chamber due to recirculation and recycling processes [12,19]. For scaling up an ED system to meet population demands, achieving the highest possible RWR is crucial, particularly in regions with limited access to water sources (e.g., coastal zones). Lower recovery rates necessitate treating larger water volumes, which increases storage capacity requirements, pumping energy consumption, and the volume of concentrate discharge per unit of treated water [53].

In the context of energy consumption, Table 2 summarizes the performance of conventional ED systems. The data suggest a possible correlation between increased system efficiency and the number of membrane pairs or stack dimensions. Patel et al. reported that the number of membrane pairs influences SEC, mitigating the effects of redox reactions at the electrodes due to changes in the separation between them [12]. For example, a comparative analysis revealed that an ED system with a single membrane pair exhibited an SEC of 1.0 kWh m^−3^, whereas a system with 50 membrane pairs reduced this to 0.1 kWh m^−3^. Similarly, Wright et al. reported SEC values of 1.1 kWh m^−3^ in a stack with 14 membrane pairs (0.2 m^2^ effective area) compared to 0.7 kWh m^−3^ in a commercial-scale system with 56 membrane pairs (37.1 m^2^ effective area) [54]. Regarding pilot-scale systems, Uche et al. obtained an SEC of 1.2 kWh m^−3^ for a system with 10 membrane pairs in a 2.5 L capacity tank, while [30] Fatima et al. assessed an ED system with 10 membrane pairs, obtaining SEC values between 1 and 2 kWh m^−3^ for an EC range of 3200–6250 µS cm^−1^ [29]. These findings align with the results of the present study, where an ED system with six membrane pairs (0.08 m^2^) achieved an SEC of 1.0 kWh m^−3^, demonstrating that ED technology is scalable and that SEC improvement can be achieved through membrane configuration optimization.

These insights into membrane configuration and system scale highlight opportunities for optimizing SEC in ED processes. However, beyond energy efficiency, environmental considerations also play a critical role in evaluating the feasibility of ED systems. Briefly, the electrical conductivity values of the concentrated brine ranged from 8.78 to 13.74 mS cm^−1^ for RWR of 43% and 66%, respectively. The discharge of this brine can disrupt marine ecosystems by affecting the osmotic balance of aquatic organisms. Nevertheless, studies have shown that its controlled release into seawater is feasible since its conductivity is considerably lower than that of typical seawater (50–70 mS/cm). This salinity gradient (≥40:1 dilution) limits environmental impacts to safe levels (<2% local salinity variation) and entails operational costs ranging from 0.05 to 0.30 USD/m^3^ [55].

**Table 2:** Energy consumption of conventional ED system: Impact of membrane pairs and effective membrane area on SEC based on literature reports.

Membrane Pairs	Effective Membrane Area (m^2^)	Feed EC µS cm^−1^	Water Sample	SEC kWh m^−3^	Reference
10	0.02	4800	Synthetic	1.24	[21]
10	0.02	3200–6250	Synthetic	1–2	[21]
14	0.18	<4800	Synthetic	1.13	[12]
50	0.34	4800	Synthetic	0.50	[21]
50	0.34	7800	Synthetic	0.75	[12]
56	37.10	<4800	Synthetic	0.73	[56]
6	0.08	3000	Groundwater	1.05	This study

Energy losses in ED systems are attributed to a couple of factors. For example, several authors have highlighted that a portion of the applied current is ineffective due to phenomena such as back diffusion and co-ion transport, which result from the non-ideal selectivity of the membranes [20,53,57]. Galama et al. emphasized that process efficiency can be negatively affected by water transport, primarily due to osmotic pressure differences between the diluted and concentrated compartments, where ions migrate through the membrane along with their hydration spheres. Consequently, the proportional transport of water across the membrane prevents a net reduction in ion concentration in the diluted stream, where the ion transport from the diluted to the concentrated compartment is not significant compared to the water transport phenomena occurring in those streams [53]. Losses in electrodialysis arise mainly from membrane non-ideal permselectivity (95%), co-ion leakage (1% of the ion flow), and ohmic resistance in the feed solution and electrodes. Although not quantified in this study, these mechanisms are well recognized and should be addressed in future optimization efforts [44,46]. Moreover, most of the losses are associated with the system’s resistance. Therefore, the use of next-generation membranes can reduce these losses, as they exhibit lower resistance and improved permselectivity. Interestingly, the impact of IEM’s characteristics on the ED process performance has been extensively studied in the literature. IEM’s thickness, water permeation, and ion exchange capacity have shown fundamental in decreasing the SEC and the resistance of the stack, as well as enhancing the kinetics of the process [58].

#### 3.2.3. Influence of Water Recovery and Ionic Composition on Specific Energy Consumption

**Figure 6:**
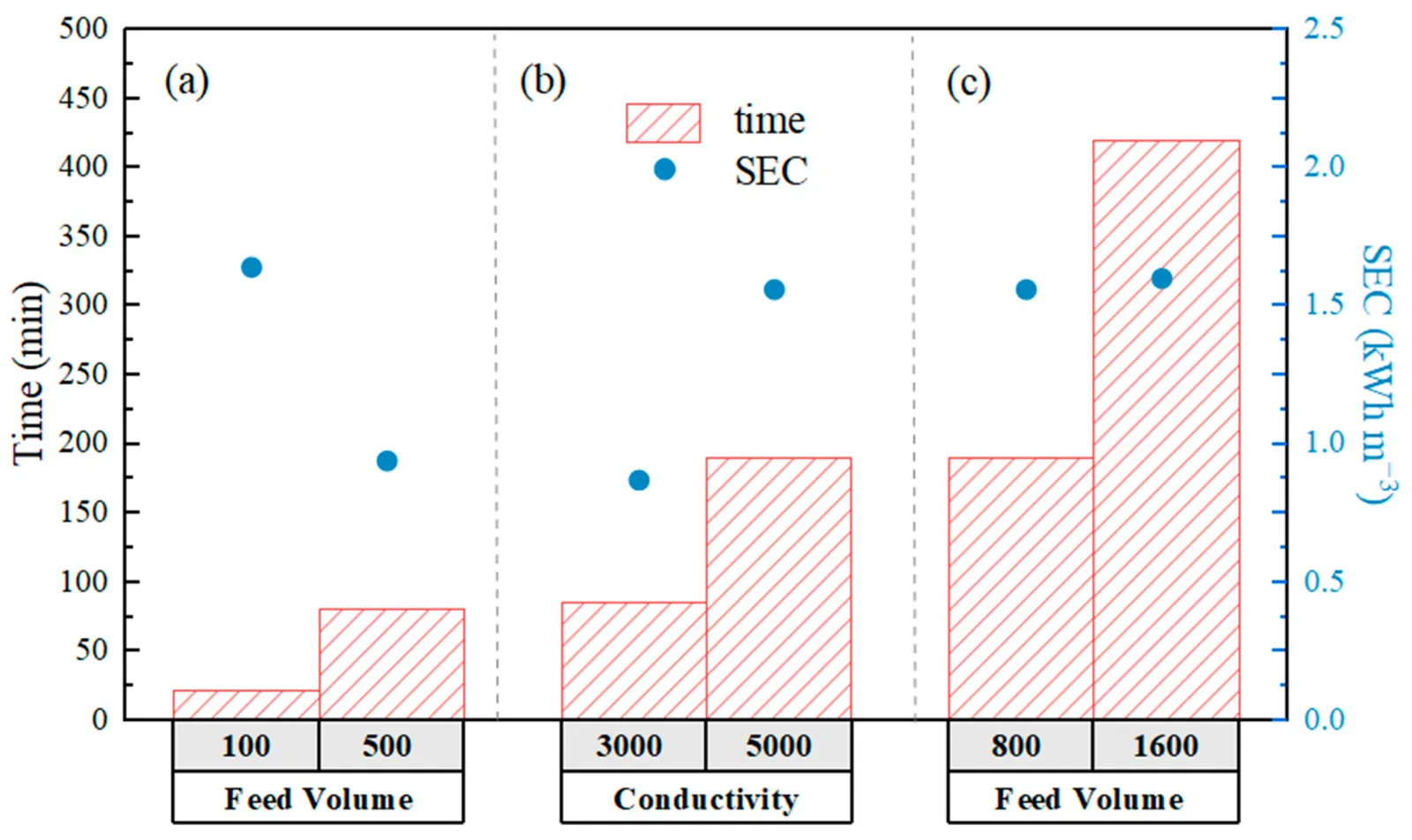
Scalability assessment of ED performance. (**a**) Effect of increasing the diluted volume at a TWR of 50% on desalination time and SEC, with an initial EC of 5000 µS cm^−1^, a cross-flow velocity of 1.3 cm s^−1^, and applied voltage of 6 V. (**b**) Effect of increasing EC on desalination time and SEC, with a diluted and concentrated volume of 500 (TWR of 50%) and a cross-flow velocity of 1.3 cm s^−1^. (**c**) Effect of increasing the diluted volume at TWR of 80% on desalination time and SEC with an EC of 5000 µS cm^−1^, an applied voltage of 6 V, and a cross-flow velocity of 1.3 cm s^−1^.

Figure 6a presents the effect of increasing the diluted volume on desalination kinetics and SEC at RWR of 43%. At an applied voltage of 6 V, the desalination time increased from 22 to 80 min as the volume increased from 100 to 500 mL. Similarly, at 8 V, the desalination time increased from 10 to 75 min, yielding time ratios of 1:4 and 1:8 for 6 V and 8 V, respectively. Processing larger volumes in batch mode resulted in longer desalination times and lower SEC values compared to smaller volumes. Specifically, increasing the volume from 100 to 500 mL led to 50% and 20% reductions in SEC at 6 and 8 V, respectively. Vera et al. reported a similar trend, where SEC decreased by 60% when comparing a bench-scale system treating 200 mL with a pilot-scale system processing 1.8 L [59]. This decrease can be attributed to an improved ion migration efficiency associated with increased volume, which reduces the energy required for ion transport.

Figure 6b illustrates the impact of increasing the EC of the feedwater samples on desalination time. As EC increased from 3000 to 5000 µS cm^−1^, the operation time doubled, rising from 80 to 190 min. Similarly, SEC increased almost two-fold, from 0.9 to 1.6 kWh m^−3^ at 6 V. Various studies have evaluated the effect of feed concentration on operating time, salt removal, and SEC. For example, Nguyen et al. reported that increasing EC from 4800 to 11,000 µS cm^−1^ led to a two-fold increase in operating time, from 30 to 55 min [23]. Similarly, Banasiak et al. observed that increasing EC from 7800 to 16,000 µS cm^−1^ doubled the operation time from 40 to 80 min [24]. Regarding SEC, Patel et al. found that increasing EC from 1600 to 16,000 µS cm^−1^ increased SEC from 0.1 to 1.8 kWh m^−3^, respectively. Higher feed concentrations require stronger driving forces due to the increased number of ions transported through the IEMs, resulting in higher SEC values [21]. Additionally, high salinity levels can induce concentration polarization [26], increase potential drops due to high ionic strength, and increase co-ion transport to maintain electroneutrality within the EIMs [21,60]. Furthermore, higher feed concentrations can impact membrane selectivity and resistance by reducing ion transport numbers [61,62].

Figure 6c illustrates the effect of increasing the diluted volume on desalination time and SEC at 66% RWR. As the volume increased from 800 to 1600 mL, the desalination time increased from 190 to 400 min, while SEC remained nearly constant at 1.56 and 1.60 kWh m^−3^, respectively. The cross-flow velocity was maintained at a constant 1.3 cm s^−1^ in the channels. This result suggests that system scalability is viable, as doubling the treated volume resulted in only a marginal increase in SEC. Consequently, increasing volume does not appear to have a detrimental impact on SEC, supporting the feasibility of scaling up the electrodialysis process.

#### 3.2.4. Effect of the Diluted Volume and Cross-Flow Velocity

**Figure 7:**
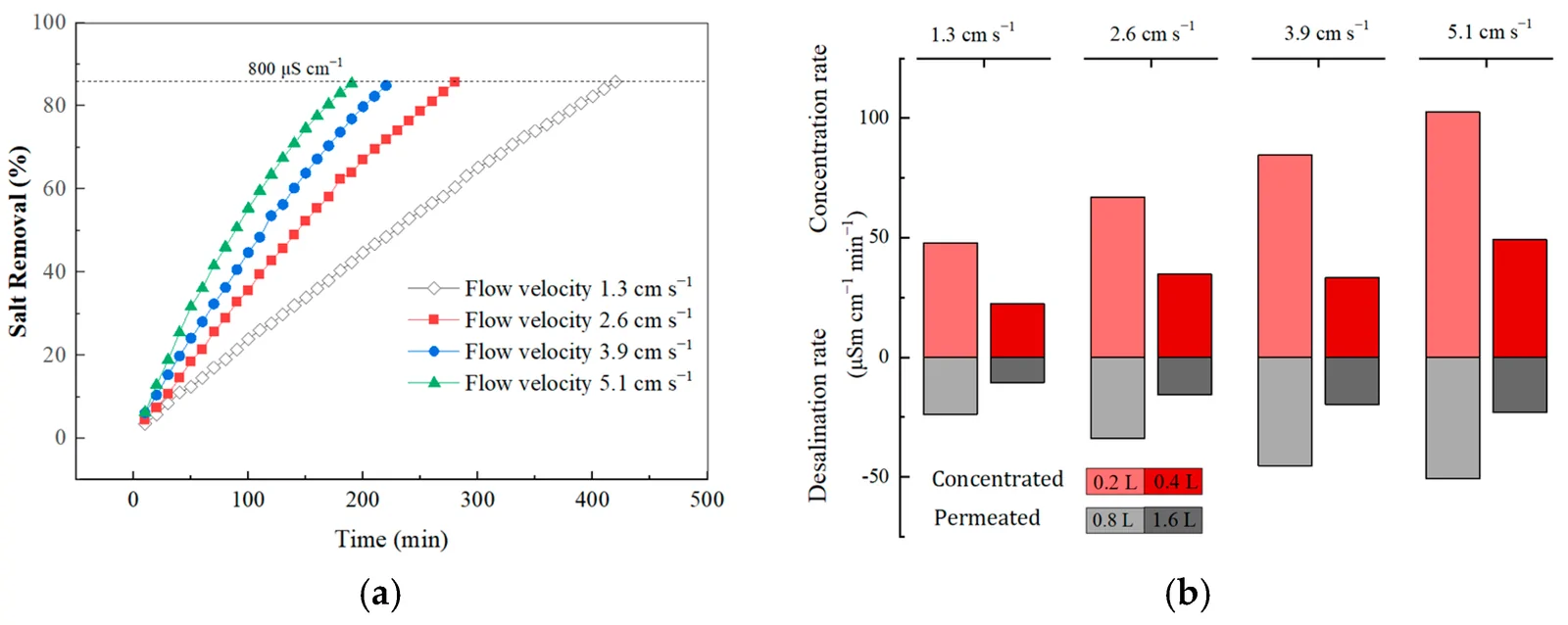
Scalability assessment of ED performance. (**a**) Effect of cross-flow velocity on desalination time to reach <800 µS cm^−1^, with a diluted volume of 1600 mL (at 80% TWR) (95% confidence and prediction bands shown in Figure A1), an initial EC of 5000 µS cm^−1^, and an applied voltage of 6 V. (**b**) Effect of diluted volume and cross-flow velocity on concentration and desalination rate, with an EC of 5000 µS cm^−1^, a TWR of 80 %, and an applied voltage of 6 V.

A representative groundwater sample of 1600 mL with an EC of 5000 µS cm^−1^ was selected, as EC had the most significant effect on SEC and better represented the scalability conditions. Figure 7a illustrates the effect of increasing cross-flow velocity on salt removal efficiency and desalination time at an applied voltage of 6 V. The results show that higher cross-flow velocities led to shorter desalination times. For example, at a velocity of 1.3 cm s^−1^ (i.e., inducing a pressure drop of ~0.3 bar), the system reached an EC of 800 µS cm^−1^ after 420 min of operation, corresponding to 85% salt removal. Increasing the flow velocity to 3.9 cm s^−1^ (i.e., pressure drop of ~0.4 bar) shortened the desalination time to 220 min, representing a ~50% reduction in desalination kinetics. Similar trends have been reported in the literature. To illustrate, He et al. observed a decrease in desalination time from 60 to 42 min in a pilot-scale system (initial EC of 1500 µS cm^−1^) when the flow rate increased from 13,000 to 22,000 mL min^−1^. Further increasing the flow rate to 25,000 mL min^−1^ resulted in a 30–40% reduction in desalination time [63]. The influence of flow velocity on desalination time is associated with enhancements in mass transfer. Wright et al. demonstrated through simulations that higher velocities increase the mass transfer coefficient, thereby accelerating the desalination rate [54]. In addition, previous investigations have demonstrated that a high flow rate influences boundary layer thickness, thereby reducing concentration polarization by promoting flow turbulence within the system [51,64,65].

Figure 7a illustrates that increasing the flow velocity from 3.9 to 5.1 cm s^−1^ (i.e., the latter producing a ~0.45 bar pressure drop) reduced the desalination time from 230 to 200 min (i.e., a 30 min reduction). This effect may be attributed to thermodynamic limitations inherent to the system. Consistent results have been reported by Demicioglu et al. during batch experiments, where varying the flow rate from 600 to 1800 mL min^−1^ resulted in a decrease in desalination time from 37 to 30 min [37]. Furthermore, Almadani treated a solution with an EC of 4800 µS cm^−1^ and found that a flow rate of 780 mL min^−1^ led to a 30% reduction in operating time compared to 150 mL min^−1^ [27]. A marginal change in desalination time at higher velocities has been attributed to reduced ion contact time across the membranes [64].

Figure 7b presents desalination and concentration rates as a function of increasing flow velocity. For a diluted volume of 800 mL (63% RWR), the desalination rates increased with higher flow velocity, with values increasing from −23.8, −33.8, −45.4, and −50.9 µS min^−1^ cm^−1^ at 1.3, 2.6, 3.9, and 5.1 cm s^−1^, respectively. Similarly, the concentration rate increased from 47.9 to 102.6 µS min^−1^ cm^−1^. Doubling the volume to 1600 mL resulted in a proportional decrease in both concentration and desalination rates. Specifically, the desalination rates at 1.3, 2.6, 3.9, and 5.1 cm s^−1^ were −10.6, −15.7, −19.7, and −23.1 µS min^−1^ cm^−1^, while the concentration rates were 22.5, 34.7, 33.2, and 49.1 µS min^−1^ cm^−1^, respectively. This reduction is not necessarily detrimental, as indicated by SEC calculations. Comparing the 800 mL and 1600 mL systems highlights advantages in energy consumption. At a flow velocity of 5.1 cm s^−1^, the SEC was 1.6 kWh m^−3^ for a diluted volume of 800 mL, whereas for 1600 mL, it was 1.5 kWh m^−3^. These results confirm that processing larger volumes does not negatively impact SEC. Additionally, the SECp was calculated based on the recorded pressure drop and flow rates, ranging between 0.033 and 0.049 kWh m^−3^, which were lower than those above observed SEC values (i.e., SEC accounts solely for electrochemical specific energy consumption). These SECp values were similarly low to those previously reported in the literature for ED systems [66] due to the low pressure drop commonly recorded in this type of system. Considering the low local energy cost (USD 0.092 kWh^−1^), pumping costs would add between USD 0.003 and USD 0.005 m^−3^, making this technology energetically favorable for the communities mentioned in this study.

#### 3.2.5. Concentrate Disposal and Reuse

The generation of concentrate as a byproduct of the desalination process presents an environmental challenge due to its high salinity, which, if not properly disposed of, can have a negative impact on the ecosystem. In this study, electrical conductivity values of the concentrated brine ranged from 8.78 to 13.74 mS cm^−1^ for RWR of 43% and 66%, respectively. There are various strategies for concentrate disposal, such as discharge into surface water bodies, deep well injection, and reuse as irrigation water [55]. However, these options must be evaluated considering operational costs and potential contamination risks. In the context of the present study, discharge into surface water bodies could be a viable option, since the site is located in a coastal area. Nevertheless, it is essential to ensure that the receiving body has a dilution capacity of at least 40 times to avoid negative impacts on marine life [67]. Also, a promising technological alternative for concentrate management is the use of bipolar membrane electrodialysis, which enables water dissociation for the production of useful acids and bases, thus adding value to the byproduct and reducing its environmental impact [68].

## 4. Conclusions

This study discusses the experimental results of batch tests performed with a laboratory-scale ED stack for desalination purposes. It was found that voltage, flow velocity, and water recovery parameters increase the salt removal efficiency. However, there are limitations, as increasing these parameters will not benefit the system, either due to phenomena such as polarization or energy increases. The design parameters that were optimal for the ED system were an applied voltage of 6 V, a water recovery of 66%, and a cross-flow velocity of 5.14 cm s^−1^, as they achieved high salt removal at the lowest energy consumption. In addition, the scalability of the system was assessed, considering the potential application of ED technology in rural or semi-arid communities. The experimentation reveals that increasing the volume from 1000 to 2000 mL leads to a threefold increase in the operational time, thus decreasing desalination rates. However, the system stands out from an energetic perspective due to the increase in productivity rates.

Considering that the system may supply drinking water to rural communities, it is ideal to operate at the highest WR since the costs associated with the water collection process would increase with the collection of a larger volume of water, thereby raising the total cost of the treatment system. Moreover, it is worth noting that stored water is generally limited in groundwater sources, unlike systems that source water from the sea. Technologies with WR less than 50%, such as RO, are not suitable for rural communities. Therefore, the experimental results of the ED system are promising, as they achieved 85% salt removal with a 66% RWR, an SECt of approximately 1.65 kWh m^−3^, and a cost of 0.15 USD m^−3^, considering the pumping energy. Nevertheless, the proposed ED system can be further improved by employing next-generation ion-exchange membranes, which exhibit lower thickness, water transport, and electrical resistance, as well as higher ion permselectivity, thereby potentially reducing energy losses and enhancing overall system performance. This study is one of the pioneering research endeavors in Ecuador, utilizing ED technology for desalinating water intended for human consumption in compliance with the Ecuadorian NTE INEN 1108 Technical Standard, which establishes the quality requirements for drinking water for human consumption. Future efforts should focus on optimizing system variables to enable scaling and pilot testing while evaluating variables that can be optimized.

## Figures and Tables

**Table 1:** Physicochemical parameters of water samples measured in the field.

Item	Sample Type	Code	pH	EC (µS cm^−1^)	TDS (mg L^−1^)	Turbidity (NTU)
1	Groundwater	P3	7.7	3070	1471	1.2
2	Groundwater	P4	7.9	1219	564	0.4
3	Groundwater	P5	7.5	2890	1410	0.6
4	Groundwater	P6	8.5	1916	969	2.5
5	Channel	P7	7.2	136	65	7.5
6	Channel	P8	7.7	146	70	4.8
7	Channel	P9	7.3	109	49	10.4
8	Surface water	P1	7.7	3250	1709	4.1
9	Surface water	P2	7.8	2010	1039	3.5

**Figure A1:**
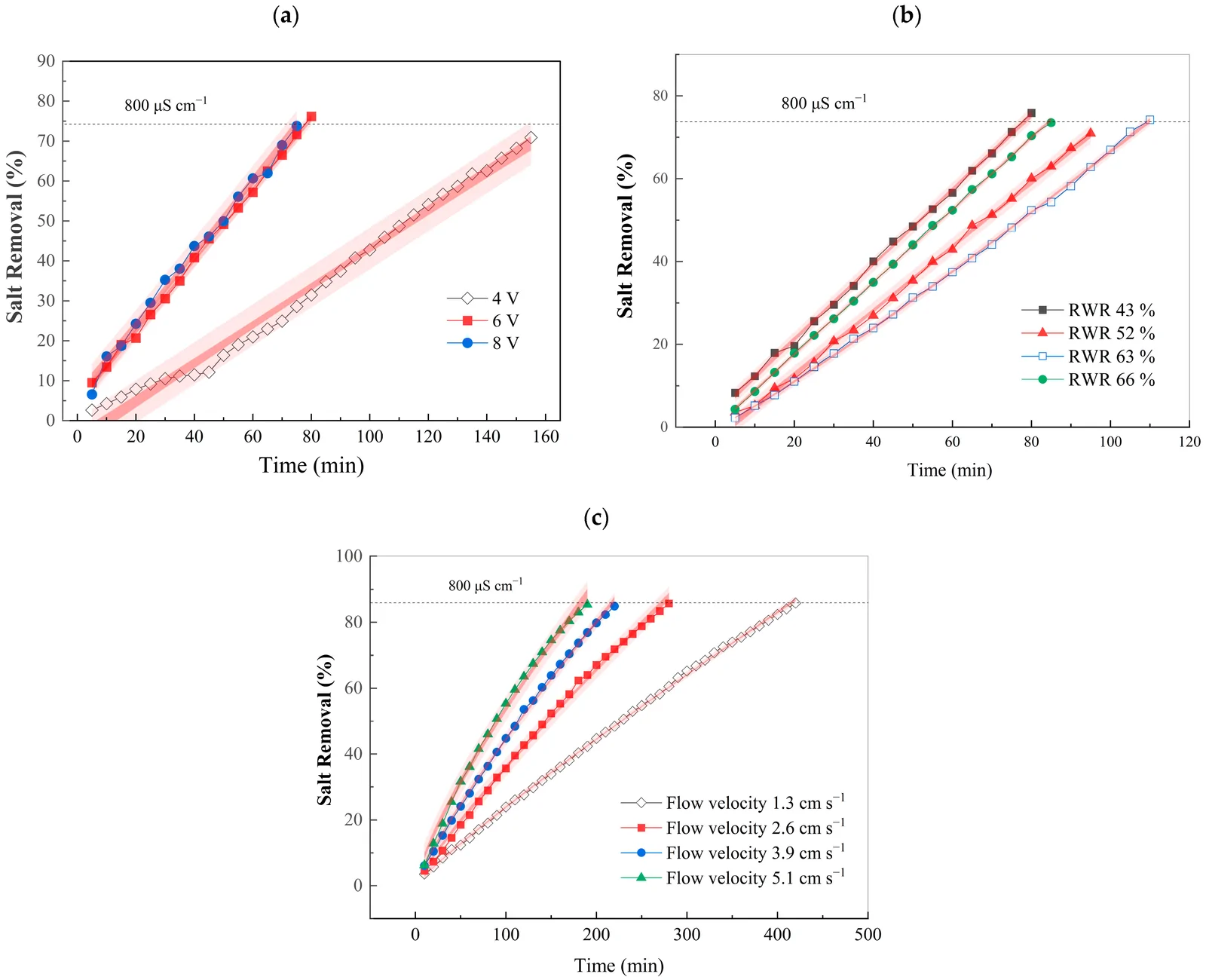
Ninety-five percent confidence and prediction bands (i.e., red and pink bands, respectively) on: (**a**) Effects of voltage on salt removal efficiencies (%) and desalination kinetics (min), (**b**) Effect of RWR on desalination time and salt removal efficiency, (**c**) Effect of cross-flow velocity on desalination time to reach <800 µS cm^−1^, with a diluted volume of 1600 mL (at 80% TWR), an initial EC of 5000 µS cm^−1^, and an applied voltage of 6 V.

## Data Availability

The original contributions presented in this study are included in the article. Further inquiries can be directed to the corresponding author.

## Abbreviations

The following abbreviations are used in this manuscript:

TWR	Theoretical Water Recovery
RWR	Real Water Recovery
TDS	Total Dissolved Solids
EC	Electrical Conductivity
WHO	World Health Organization
SEC	Specific Energy Consumption
RO	Reverse Osmosis
ED	Electrodialysis
CEM	Cation-Exchange Membranes
AEM	Anion-Exchange Membranes
IEM	Ion-Exchange Membrane
SEC	(Electrochemical) Specific Energy Consumption
SECp	Specific Energy Consumption of Pump
SECt	Total Specific Energy Consumption
